# Special Issue: Gram-Positive Bacterial Toxins

**DOI:** 10.3390/microorganisms11082054

**Published:** 2023-08-10

**Authors:** Shashi Sharma, Sabine Pellett, Stephen A. Morse

**Affiliations:** 1 Division of Microbiology, Office of Regulatory Science, CFSAN/US Food and Drug Administration, College Park, MD 20740, USA; 2 Department of Bacteriology, University of Wisconsin, 1550 Linden Drive, Madison, WI 53706, USA; 3 IHRC, Inc., 2 Ravinia Drive, Suite 1200, Atlanta, GA 30346, USA

The Gram stain classifies most bacteria into one of two groups, Gram-negative or Gram-positive, based on the composition of their cell walls. Gram-positive bacteria are a diverse group of microorganisms that include many important human and zoonotic pathogens, including *Staphylococcus aureus*, *Streptococcus pyogenes*, *Streptococcus pneumoniae*, *Listeria monocytogenes*, *Clostridium difficile*, *Clostridium botulinum*, and *Bacillus anthracis.* Gram-positive bacteria produce a range of toxins that may contribute to their pathogenicity [1,2]. Recent research has shown that many of these toxins play an essential role in the survival and virulence of these bacteria, but for some toxins their evolutionary significance and potential benefits to the bacteria harboring them remains unclear. In this Special Issue of *Microorganisms*, we highlight recent advancements in the importance of Gram-positive bacterial toxins in infectious diseases.

Toxins are an important factor in the pathogenesis of many Gram-positive bacterial infections, causing tissue damage and contributing to the severity of disease. They can also have effects beyond their direct role in pathogenesis, such as influencing the behavior of other bacteria in the microbiome, altering the host immune response, and even susceptibility to antimicrobial agents [3]. In response to these toxins, the host immune system produces antibodies and a variety of cytokines, including interferon gamma (IFN-γ), that play a role in both protective and pathogenic responses [4]. One class of toxins, termed superantigens, are produced by some Gram-positive bacteria (e.g., *S. aureus* and *S. pyogenes*) and can cause a massive cytokine response and have been implicated in a number of diseases [5].

Several toxins produced by Gram-positive bacteria have also become a focus of research for their potential as therapeutic targets as well as therapeutic agents. Antibody-based therapeutics directed against Gram-positive bacterial toxins have been developed and are currently in preclinical or clinical trials [6,7]. Clinical uses of toxins continue to be explored and expanded, as exemplified by the many clinical applications for botulinum neurotoxins [8,9,10]. In spite of the many exciting advances in this field, much remains to be learned about the mechanisms underlying the pathogenic effects of these toxins on the host, and potential utility in medicine.

Recent studies have shed more light on some specific pathogenic effects of Gram-positive bacterial toxins on their host. For example, the Panton–Valentine leukocidin (PVL) produced by *S. aureus* has been shown to cause lung inflammation and injury, mediated in part by polymorphonuclear leukocytes [11,12,13,14]. The streptolysin S (SLS) produced by *S. pyogenes* was found to target the sodium-bicarbonate co-transporter NBCn1 in keratinocytes, resulting in NF-κB activation and the cytotoxicity of intoxicated keratinocytes, a process which increases disease severity [15]. Other studies have advanced the utility of Gram-positive bacterial toxins in the medical field. The detection of *C. difficile* toxins in stool samples has also become an important tool for the diagnosis of *C. difficile* infection [16]. Further research is needed to fully understand the role toxins play in the interaction of Gram-positive bacteria with the microbiome and how this may impact host health and wellbeing, as well as to develop new therapeutic approaches that target both the toxin and the bacteria that produce them [17].

The decision to focus on Gram-positive bacterial toxins in this Special Issue is a timely and important one. Gram-positive bacteria produce numerous toxins of great global health significance, including anthrax toxins, *Bacillus cereus* enterotoxin, diphtheria toxin, staphylococcal toxins, streptococcal toxins, tetanus toxin, botulinum toxin, pneumolysin, enterococcal toxin, and more. By publishing research papers that explore these topics, this journal can help advance our understanding of these toxins and their impact on human health.

This emphasis on Gram-positive bacterial toxins has the potential to contribute new knowledge in a number of significant areas. For example, research on these toxins could lead to the development of new therapies or vaccines that target specific bacterial toxins, ultimately improving patient outcomes. Additionally, by exploring the molecular mechanisms underlying the activity of these toxins, researchers may be able to identify new targets for drug development and gain insights into the basic biology of bacterial toxins. Finally, exploring clinical applications of these toxins as bio-therapeutics in disease management or treatment has the potential to improve patient outcome and well-being.

We acknowledge the contributors for their significant contributions to the first edition of this Special Issue. We invite scholars to contribute by submitting their most recent research findings on Gram-positive bacterial toxins for publication in the second edition of this Special Issue. The journal has extended the publication of the second Special Issue based on the outstanding response we have received. By sharing your work and insights with the wider community, we can collectively advance our understanding of these toxins and their impact on human health, ultimately leading to new and more effective treatments for bacterial infections.

## 1. Role of Some Gram-Positive Bacterial Toxins in Infectious Diseases

Exotoxins contribute significantly to the pathogenesis of diseases. These toxins are secreted by toxin-producing microorganisms and can cause damage to host tissues, leading to the development of disease. One of the most well-known Gram-positive bacterial toxins is the α-toxin of *S. aureus*, which contributes to host tissue damage and disease cause by *S. aureus*, which causes >1 million deaths per year globally [18]. α-Toxin forms pores in the host cell membranes, leading to cell lysis and tissue damage [19]. Recent studies have shown that α-toxin can also activate the inflammasome, a critical component of the innate immune system. Inflammasome activation leads to the production of cytokines, which can cause tissue damage and inflammation [20].

Another important Gram-positive bacterial toxin is the streptolysin O (SLO) of *S. pyogenes*, which has a morbidity in the millions worldwide with significant associated mortality [20,21]. SLO is known to be hemolytic and can damage host cells. SLO also contributes to the development of the inflammatory response in infected tissues. Recent studies have shown that this toxin can activate the NLRP3 inflammasome, leading to the production of cytokines and the recruitment of immune cells to the site of infection [22].

The lethal toxin (LT) produced by the potential bioweapon *B. anthracis* is also an important Gram-positive bacterial toxin [23,24]. LT is comprised of Lethal Factor (LF) and protective antigen. LF is a zinc metalloprotease that cleaves most isoforms of mitogen-activated protein kinases close to the N-terminus. In a mouse model, LF has been shown to inhibit the activation of many cells of the innate and adaptive immune systems, including PMNs, T cells, B cells, monocytes, and macrophages. Recent studies have shown that LT can activate the inflammasome, leading to the production of cytokines and the recruitment of immune cells to the site of infection [25,26].

Botulinum neurotoxins (BoNTs), another potential bioweapon, are produced by *C. botulinum* and some related clostridia and cause the potentially lethal disease botulism. BoNTs are AB-type neurotoxins that specifically enter neuronal cells, predominantly motor neurons. These toxins bind to specific neuronal cell surface receptors via the B-domain, leading to endocytosis and the translocation of the enzymatic A-domain into the cell cytosol. Inside the cell cytosol, the toxins cleave proteins essential for the fusion machinery of synaptic vesicles, thereby blocking neurotransmitter release and neuro-transmission, which leads to flaccid paralysis [27,28]. BoNTs are noteworthy in that they are also widely used as bio-therapeutics to treat a myriad of neuronal disorders [29].

## 2. Advancements in the Study of Gram-Positive Bacterial Toxins

A recent review mentions a novel toxin produced by *S. aureus* that was found to be a potent inducer of inflammation and tissue damage. This study highlights the importance of identifying new toxins and their mechanisms of action to help understand their role in the pathogenesis of infectious diseases [14].

Another area of research is the development of new therapies to target Gram-positive bacterial toxins. One promising approach is the use of monoclonal antibodies to neutralize the toxins. For example, a recent study showed that monoclonal antibodies targeting the α-toxin of *S. aureus* were effective in reducing tissue damage and inflammation in a mouse model of pneumonia. This study demonstrates the potential of monoclonal antibodies as a therapeutic approach for Gram-positive bacterial infections [30,31,32,33].

The use of bacteriophages to target Gram-positive toxin-producing bacteria is another area of research. Bacteriophages are viruses that infect and kill bacteria. Recent studies have shown that bacteriophages can be effective in reducing the bacterial load and therefore toxin production in animal models of Gram-positive bacterial infections [24]. This approach has the potential to provide a new therapeutic option for the treatment of infectious diseases caused by antibiotic-resistant Gram-positive bacteria.

## 3. Gaps in Research and Future Advances

Despite the recent advancements in the study of Gram-positive bacterial toxins, several gaps remain. One of the key challenges is the identification of new toxins and their mechanisms of action. Although significant progress has been made in this area, there are potentially many unknown toxins with unexplored effects on host tissues. Genome mining continues to identify new toxins, homologs to known toxins, and new toxin variants [34]. Structural and functional studies of these toxins to understand their mechanisms of action can provide new targets for the development of therapies.

The development of effective therapies to target Gram-positive bacteria and their toxins remains a challenge. While antibody therapies [35] and bacteriophages [36] show promise, further research is needed to expand and optimize these approaches and determine their efficacy in clinical trials. Additionally, the potential for bacterial resistance to these therapies should also be considered. Increasing our understanding of the molecular mechanisms involved in pathogenesis will undoubtedly open up new avenues for the development of additional therapies.

The interaction between Gram-positive bacterial toxins and the host immune system is a complex and multifaceted process. For instance, recent studies have shown that some Gram-positive bacterial toxins can activate the host immune system, leading to the production of cytokines and the recruitment of immune cells to the site of infection [37,38]. However, the precise mechanisms of this interaction are not well understood, and further research is needed to elucidate this process fully and develop treatment strategies.

Another area of future research is the development of new diagnostic tools for the detection of Gram-positive bacterial toxins. Current diagnostic methods for bacterial infections rely mainly on culture or nucleic acid amplification tests (e.g., PCR). Culture can take several days and may not be accurate in some cases. Currently available PCR assays do not detect protein toxins directly, although they can detect the genes encoding the toxins. New diagnostic methods capable of reliably and sensitively detecting the presence of toxins directly in patient samples would provide more rapid and accurate diagnosis, allowing for earlier treatment and better patient outcomes.

There is also a need for further research into the role of Gram-positive bacterial toxins in chronic infections. While much of the research has focused on acute infections, Gram-positive bacterial toxins have been implicated in the pathogenesis of chronic infections, such as osteomyelitis and endocarditis [37,38,39,40,41]. Understanding the mechanisms by which these toxins may contribute to chronic infections can provide new targets for the development of effective therapies to treat these persistent infections.

Finally, the role of host microbiota in the pathogenesis of infectious diseases caused by Gram-positive bacteria is an area of growing interest. Recent research has shown that alterations in host microbiota can affect the susceptibility to infection and severity of disease [42]. Further research is needed to understand the complex interplay between host microbiota, Gram-positive bacterial toxins, and host immune responses in the pathogenesis of infectious diseases.

## 4. Concluding Remarks

In conclusion, the study of Gram-positive bacterial toxins has provided significant insights into the pathogenesis of infectious diseases. However, there is still much to be learned about these toxins, including their mechanisms of action, their interactions with the host immune system, their interactions with host microbiota, and their role in chronic infections. Further basic research in these areas has the potential to provide new targets for the development of therapies to treat infectious diseases caused by Gram-positive bacteria.
